# Supplementary material for: Bioreplicated coatings for photovoltaic solar panels nearly eliminate light pollution that harms polarotactic insects
Source: PLoS One. 2020 Dec 3;15(12):e0243296. doi: 10.1371/journal.pone.0243296 (PMC7714120; doi:10.1371/journal.pone.0243296)
Supplement: S1 Fig — The polarization patterns were measured with imaging polarimetry in the blue (450 nm) spectral range when the sun shone from behind (top) and left (bottom) and light from the clear sky was reflected from the test surfaces. The tilt angle of the optical axis was -35° from the horizontal. In the middle row, the numerical values are the degrees of polarization averaged for the different test surfaces. In the angle of polarization patterns, the white bars show the average directions of polarization of the test surfaces. (DOCX) [file pone.0243296.s001.docx]

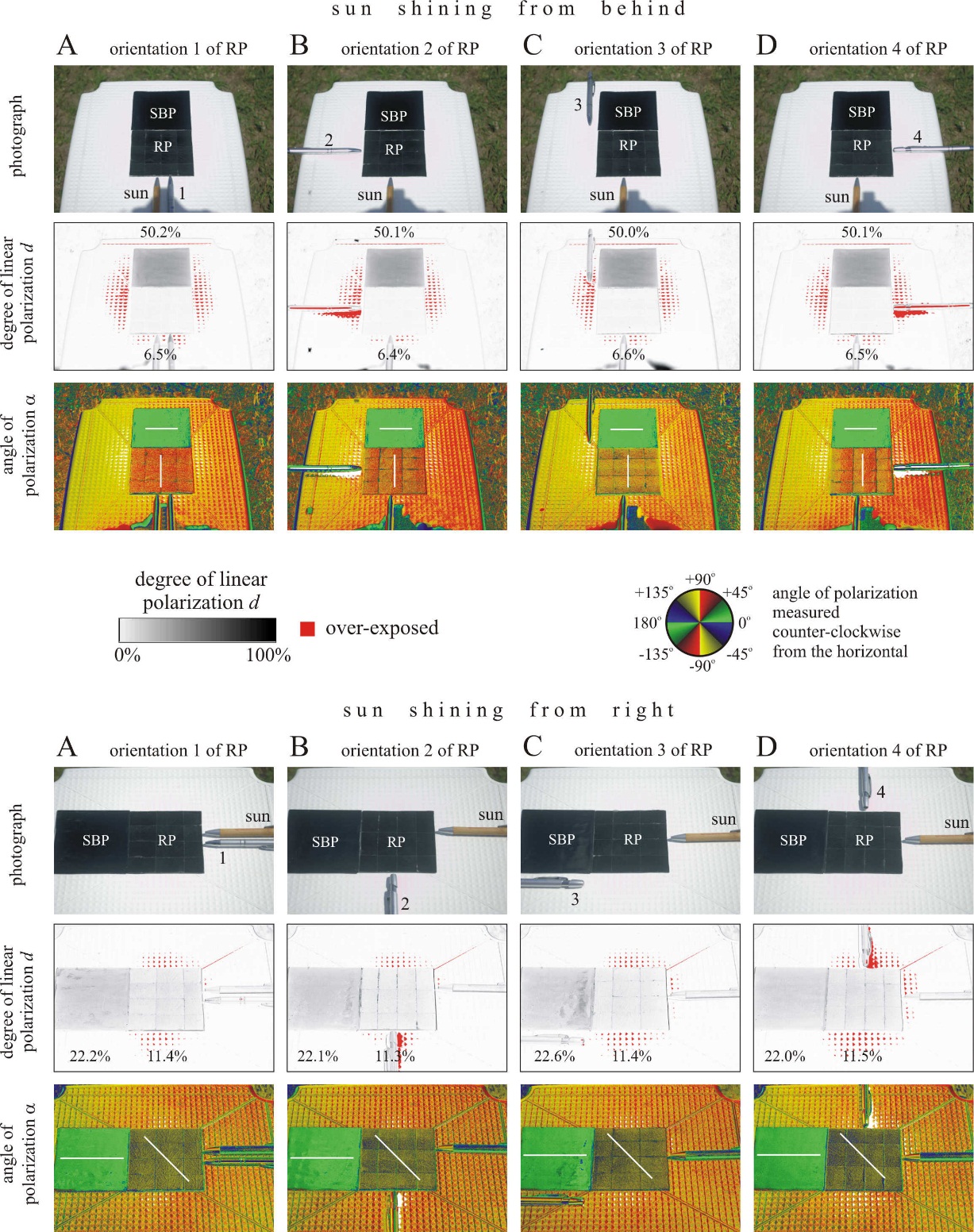


**S1 Fig.** **Photographs and patterns of the degree and angle of polarization of the rose petal (RP) replica and the planar PMMA (SBP) reference layer used in the field experiments with mayflies and horseflies for four different orientations of the RP.** The polarization patterns were measured with imaging polarimetry in the blue (450 nm) spectral range when the sun shone from behind (top) and left (bottom) and light from the clear sky was reflected from the test surfaces. The tilt angle of the optical axis was -35° from the horizontal. In the middle row, the numerical values are the degrees of polarization averaged for the different test surfaces. In the angle of polarization patterns, the white bars show the average directions of polarization of the test surfaces.
