# Supplementary material for: Bioreplicated coatings for photovoltaic solar panels nearly eliminate light pollution that harms polarotactic insects
Source: PLoS One. 2020 Dec 3;15(12):e0243296. doi: 10.1371/journal.pone.0243296 (PMC7714120; doi:10.1371/journal.pone.0243296)
Supplement: S2 Fig — The fully packed cones have an aspect ratio AR = 0.6 with standard deviations σh = σp = 0, where σh is the disorder of cone height and σp is the disorder of cone position. (DOCX) [file pone.0243296.s002.docx]

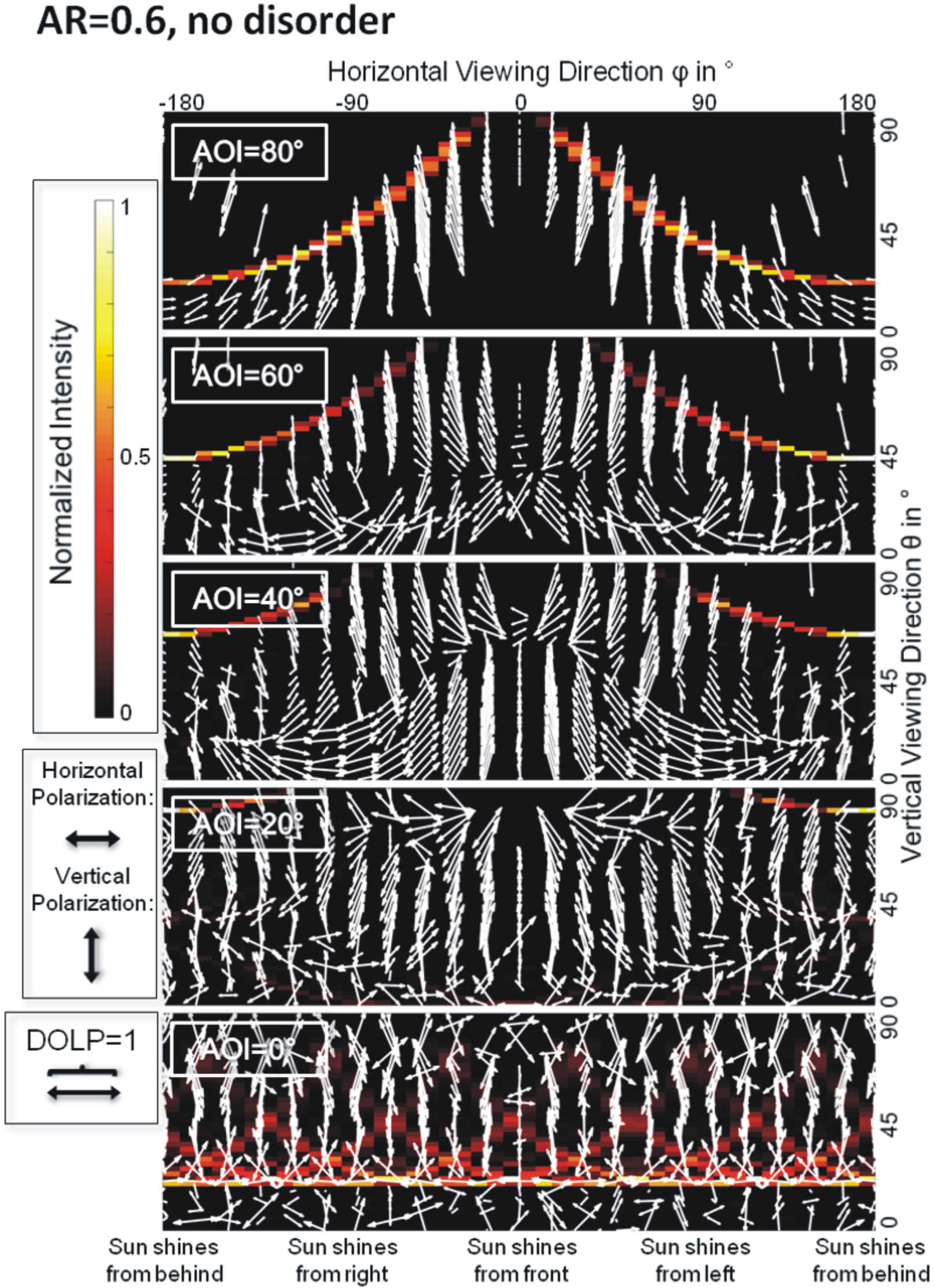


**S2 Fig. Simulated farfield reflection-polarization characteristics (light intensity shown in colours, and polarization represented by double-headed arrows, the length of which is proportional to the local degree of linear polarization DoLP) as functions of observer position and angle of incidence AoI.** The fully packed cones have an aspect ratio AR = 0.6 with standard deviations σ_h_ = σ_p_ = 0, where σ_h_ is the disorder of cone height and σ_p_ is the disorder of cone position.
