# Supplementary material for: Bioreplicated coatings for photovoltaic solar panels nearly eliminate light pollution that harms polarotactic insects
Source: PLoS One. 2020 Dec 3;15(12):e0243296. doi: 10.1371/journal.pone.0243296 (PMC7714120; doi:10.1371/journal.pone.0243296)
Supplement: S3 Fig — (DOCX) [file pone.0243296.s003.docx]

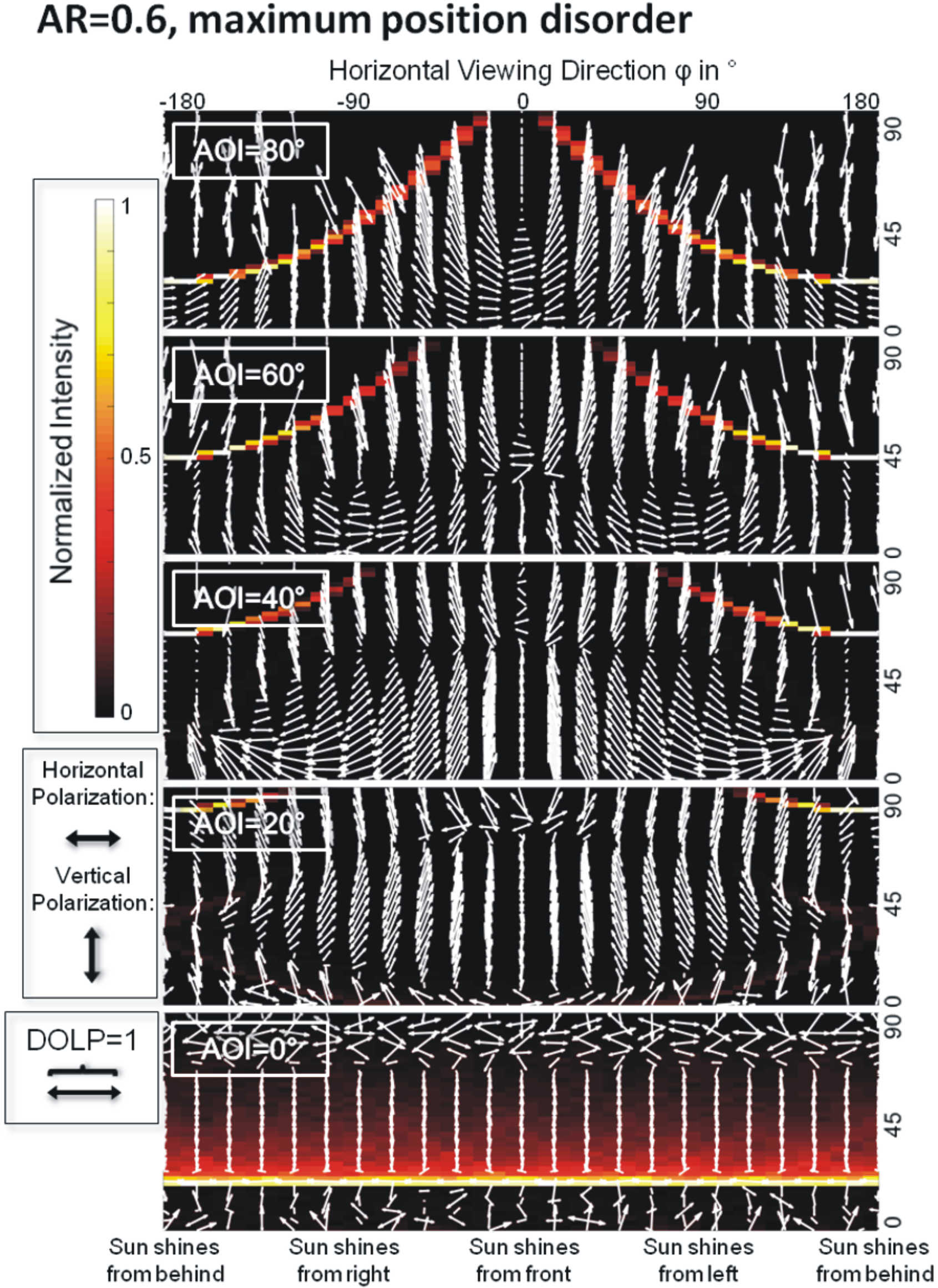


**S3 Fig.** **As S2 Fig for microcones with aspect ratio AR = 0.6, σ_h_ = 0 and σ_p_ = 0.5·d¯, where d¯ is the average distance between nearest cone neighbours for the unperturbed, hexagonally arranged model.**
