# Supplementary figures and images for: Bioreplicated coatings for photovoltaic solar panels nearly eliminate light pollution that harms polarotactic insects
Source: PLoS One. 2020 Dec 3;15(12):e0243296. doi: 10.1371/journal.pone.0243296 (PMC7714120; doi:10.1371/journal.pone.0243296)

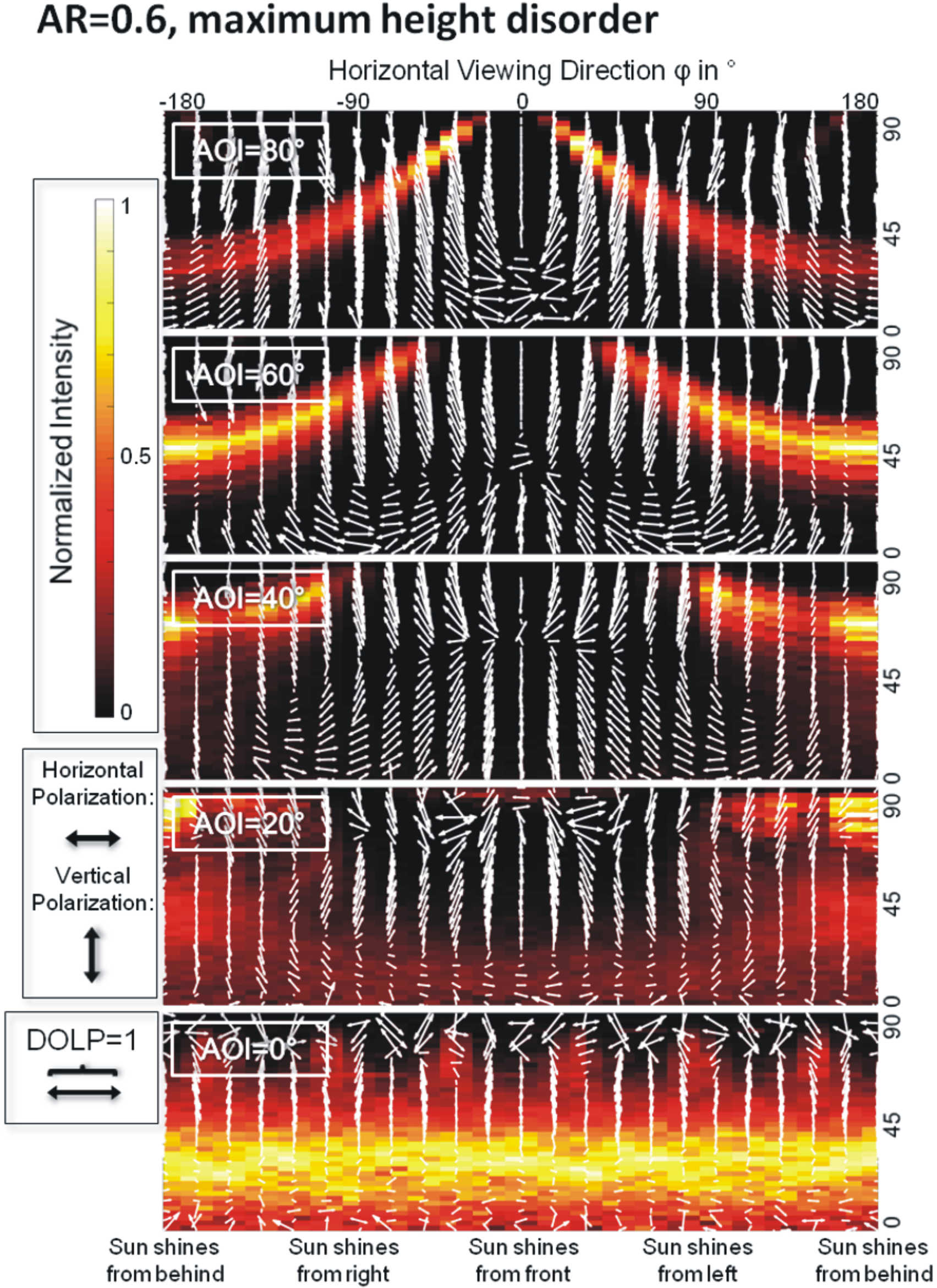


**S4 Fig. As S2 Fig for microcones with aspect ratio AR = 0.6, σh = 0.3·h¯ and σp = 0, where h¯ is the average cone height.**

Supplement: S4 Fig — (DOCX) [file pone.0243296.s004.docx]
