# Supplementary material for: Bioreplicated coatings for photovoltaic solar panels nearly eliminate light pollution that harms polarotactic insects
Source: PLoS One. 2020 Dec 3;15(12):e0243296. doi: 10.1371/journal.pone.0243296 (PMC7714120; doi:10.1371/journal.pone.0243296)
Supplement: S1 Table — The daily time period (UTC + 2 hours) of the experiment is also given. (DOCX) [file pone.0243296.s005.docx]

**S1 Table.** **Number of landings of Ephemera danica mayflies on the three different test surfaces (RP: rose petal, GRP: glass-covered rose petal, SBP: smooth black plastic) used in the field experiments on 6, 7, 10, 11, 12 and 13 June 2019.** The daily time period (UTC + 2 hours) of the experiment is also given.

| **date**  **2019** | **time period**  **(UTC+2 h)** | **RP** | **GRP** | **SBP** |
| --- | --- | --- | --- | --- |
| **6 June** | 19:45-20:00 | 0 | 4 | 4 |
| **6 June** | 20:00-20:15 | 0 | 3 | 6 |
| **6 June** | 20:15-20:30 | 0 | 2 | 2 |
| **6 June** | 20:30-20:45 | 0 | 1 | 3 |
| **6 June** | 20:45-21:00 | 0 | 2 | 4 |
| **7 June** | 19:15-19:30 | 0 | 10 | 12 |
| **7 June** | 19:30-19:45 | 0 | 8 | 12 |
| **7 June** | 19:45-20:00 | 1 | 12 | 16 |
| **7 June** | 20:00-20:15 | 0 | 15 | 32 |
| **7 June** | 20:15-20:30 | 0 | 8 | 18 |
| **7 June** | 20:30-20:45 | 0 | 3 | 9 |
| **7 June** | 20:45-21:00 | 0 | 13 | 9 |
| **10 June** | 19:00-19:15 | 0 | 3 | 14 |
| **10 June** | 19:15-19:30 | 0 | 0 | 7 |
| **10 June** | 19:30-19:45 | 0 | 6 | 13 |
| **10 June** | 19:45-20:00 | 0 | 14 | 23 |
| **10 June** | 20:00-20:15 | 8 | 11 | 12 |
| **10 June** | 20:15-20:30 | 1 | 18 | 6 |
| **10 June** | 20:30-20:45 | 0 | 2 | 2 |
| **10 June** | 20:45-21:00 | 11 | 59 | 19 |
| **11 June** | 18:30-18:45 | 1 | 22 | 33 |
| **11 June** | 18:45-19:00 | 0 | 21 | 46 |
| **11 June** | 19:00-19:15 | 1 | 6 | 10 |
| **11 June** | 19:15-19:30 | 0 | 16 | 16 |
| **11 June** | 19:30-19:45 | 0 | 1 | 6 |
| **11 June** | 19:45-20:00 | 1 | 4 | 10 |
| **11 June** | 20:00-20:15 | 0 | 13 | 20 |
| **11 June** | 20:15-20:30 | 0 | 8 | 9 |
| **11 June** | 20:30-20:45 | 3 | 17 | 25 |
| **11 June** | 20:45-21:00 | 1 | 12 | 23 |
| **12 June** | 18:30-18:45 | 0 | 11 | 14 |
| **12 June** | 18:45-19:00 | 1 | 8 | 11 |
| **12 June** | 19:00-19:15 | 0 | 9 | 9 |
| **12 June** | 19:15-19:30 | 0 | 5 | 7 |
| **12 June** | 19:30-19:45 | 0 | 5 | 10 |
| **12 June** | 19:45-20:00 | 0 | 3 | 5 |
| **12 June** | 20:00-20:15 | 0 | 1 | 3 |
| **12 June** | 20:15-20:30 | 0 | 13 | 14 |
| **12 June** | 20:30-20:45 | 1 | 20 | 18 |
| **12 June** | 20:45-21:00 | 2 | 17 | 19 |
| **13 June** | 18:45-19:00 | 0 | 10 | 13 |
| **13 June** | 19:00-19:15 | 0 | 5 | 8 |
| **13 June** | 19:15-19:30 | 0 | 4 | 9 |
| **13 June** | 19:30-19:45 | 0 | 2 | 2 |
| **13 June** | 19:45-20:00 | 0 | 2 | 4 |
| **13 June** | 20:00-20:15 | 1 | 6 | 10 |
| **13 June** | 20:15-20:30 | 0 | 3 | 9 |
| **13 June** | 20:30-20:45 | 1 | 8 | 11 |
| **13 June** | 20:45-21:00 | 0 | 7 | 5 |
| **sum** |  | **23** | **394** | **583** |
