# Supplementary material for: Bioreplicated coatings for photovoltaic solar panels nearly eliminate light pollution that harms polarotactic insects
Source: PLoS One. 2020 Dec 3;15(12):e0243296. doi: 10.1371/journal.pone.0243296 (PMC7714120; doi:10.1371/journal.pone.0243296)
Supplement: S2 Table — The daily time period (UTC + 2 hours) of the experiment is also given. (DOCX) [file pone.0243296.s006.docx]

**S2 Table.** **Numbers of landings of horseflies on the three different test surfaces (RP: rose petal, GRP: glass-covered rose petal, SBP: smooth black plastic) used in the field experiment on 18, 19, 25 and 27 June 2019.** The daily time period (UTC + 2 hours) of the experiment is also given.

| **date**  **2019** | **time period**  **(UTC+2 h)** | **RP** | **GRP** | **SBP** |
| --- | --- | --- | --- | --- |
| 18 June | 9:30-10:00 | 0 | 3 | 1 |
| 18 June | 10:00-10:30 | 2 | 5 | 11 |
| 18 June | 10:30-11:00 | 1 | 3 | 10 |
| 18 June | 11:00-11:30 | 1 | 17 | 12 |
| 18 June | 11:30-12:00 | 0 | 6 | 7 |
| 18 June | 12:00-12:30 | 1 | 12 | 0 |
| 18 June | 12:30-13:00 | 0 | 4 | 2 |
| 19 June | 9:30-10:00 | 0 | 0 | 1 |
| 19 June | 10:00-10:30 | 0 | 2 | 0 |
| 19 June | 10:30-11:00 | 0 | 6 | 0 |
| 19 June | 11:00-11:30 | 1 | 2 | 1 |
| 19 June | 11:30-12:00 | 0 | 7 | 0 |
| 25 June | 10:30-11:00 | 0 | 1 | 0 |
| 25 June | 11:00-11:30 | 0 | 0 | 1 |
| 25 June | 11:30-12:00 | 1 | 0 | 3 |
| 25 June | 12:00-12:30 | 0 | 5 | 0 |
| 25 June | 12:30-13:00 | 0 | 0 | 3 |
| 25 June | 13:00-13:30 | 0 | 6 | 0 |
| 25 June | 13:30-14:00 | 0 | 0 | 10 |
| 27 June | 9:30-10:00 | 0 | 2 | 0 |
| 27 June | 10:00-10:30 | 0 | 5 | 0 |
| 27 June | 10:30-11:00 | 0 | 0 | 0 |
| 27 June | 11:00-11:30 | 0 | 0 | 3 |
| 27 June | 11:30-12:00 | 0 | 6 | 0 |
| 27 June | 12:00-12:30 | 0 | 1 | 7 |
| 27 June | 12:30-13:00 | 0 | 1 | 0 |
| 27 June | 13:00-13:30 | 0 | 18 | 0 |
| 27 June | 13:30-14:00 | 0 | 0 | 10 |
| **sum** |  | **7** | **112** | **82** |
