# Supplementary material for: Bioreplicated coatings for photovoltaic solar panels nearly eliminate light pollution that harms polarotactic insects
Source: PLoS One. 2020 Dec 3;15(12):e0243296. doi: 10.1371/journal.pone.0243296 (PMC7714120; doi:10.1371/journal.pone.0243296)
Supplement: S1 File — (DOCX) [file pone.0243296.s007.docx]

**Ray tracing simulations**

**Convergence tests and simulation model dimensions**

To determine the appropriate minimum number *N*_min_ of rays necessary to reliably calculate the dimensions, geometrical parameters and other relevant features of our 3D microtexture model, convergence tests were carried out for the whole range of investigated geometries and adapted for all intermediate steps that led to our final geometrical configuration. During these tests the number *N* of rays were increased up to *N*_min_ when the reflectance began to converge toward a limit for all angle of incidences considered. A further increase of *N* would have resulted in only the increase of the computation time without a significant change of the resulting reflectance. For our final models, *N*_min_ = 10^8^ rays were sufficient to produce reliable results for all cone dimensions and degrees of disorder considered herein.

When simulating the interaction of light with a disordered surface texture, a large enough number of structural units (here microcones) has to be included into the simulation window to achieve reliable and statistically relevant results. In particular, repeating a calculation with a different 3D model which was built from the same geometrical parameters (including the disorder of cone height σ_h_ > 0 and the disorder of cone position σ_p_ > 0) should lead to almost identical results. Such tests were performed and held true for all simulated properties including the total amount of power being reflected, the reflected farfield intensity distribution, the farfield distribution of the degree and angle of linear polarization. For a certain angle of incidence, the light source was always moved to the side such that the rays always illuminate the same central area of the microtexture model. If one would choose to illuminate the full area of the 3D texture models, then an increasing number of rays would be directly incident on the mirrors surrounding the texture model and therefore change propagation direction before interacting with the cones. To avoid this distortion of the reflected light’s farfield distribution, all 3D microtexture models were built with enough cones to 1) illuminate a large enough number of them to properly incorporate disorder and 2) still leave enough space around the illuminated part to always have less than 1% of the total power emitted by the light source interacting with the mirrors around the texture. Limited by the fact that the required computation time increases exponentially with increasing number of geometrical features of the 3D model, these considerations led to a configuration of the microcone models with 753 cones in total and 419 illuminated cones, as depicted in Fig 3.

**Microtexture models**

An aspect ratio of the microcones between 0.3 and 1.0 in 0.1 steps was considered in a "full tiling" configuration, meaning that nearest neighbors overlap such that there the whole rectangular base is covered with microcones. A planar rectangular (and transparent) light source emitted 10^8^ parallel and unpolarized rays of identical initial power with random starting positions into the central part of the microtexture models (Fig 3A,B). For angle of incidence > 0°, the light source was moved to the (φ, θ) = (180°, 90°) direction (Fig 3A,B) such that the illuminated part of the texture is kept constant. angle of incidences between 0° and 80° in 20° steps were investigated. Structural disorder in microcones height and in their arrangement (in-plane position) was incorporated into the ray tracing models by introducing two standard deviations, the disorder σ_h_ of cone height, and the disorder σ_p_ of cone position. For σ_p_ = 0, all cones are placed onto the planar base according to a hexagonal array. If σ_p_ > 0 is chosen, a lateral displacement value, which is taken from a gaussian distribution with standard deviation σ_p_ and an average of zero, is randomly assigned to every cone. This way, every cone will be displaced along an arbitrary lateral direction. Furthermore, every cone will be assigned a certain height. The values are taken from a normal distribution with standard deviation σ_h_ and a user-defined average height of *h*¯. For all the simulations that were performed during this study, the cone radius was kept constant at 10 µm and therefore, disorder in height can be directly translated into disorder in AR. More details about the modeling algorithm for such disordered microtextures can be found in Reference (1).

Disorder in cone height was increased from σ_h_ = 0 to σ_h_ = 0.3·h¯ in 0.1·h¯ steps. For the disordered arrangement of the microcones we chose to ramp up σ_p_ from zero to 0.5·d¯ in 0.125·d¯ steps. Here h¯ and d¯ are the average cone height and average distance between nearest neighbours for the unperturbed, hexagonally arranged model. The range for σ_h_ well incorporates the natural height variations found in the conical epidermal cells of petal surface textures (see Fig 1B-C). Moreover, the maximum value chosen for σ_p_ leads to a fully random cone arrangement [1). Congruently to the samples we used for our field studies, the optical parameters of PMMA were implemented as the material properties of the ray tracing models. As for the experimentally obtained polarization properties of reflected light presented in this communication, a wavelength of 450 nm (and therefore a refractive index of n_PMMA_ = 1.49) was chosen for all simulations described in this paragraph. If a ray intersects the surface of one of the cones, the Fresnel equations are used to calculate the probabilities for reflectance and transmittance for the local angle of incidence, wavelength and polarization state of the light ray.

**Simulated reflection-polarization characteristics and graphical display**

For the data display of the farfield distribution of reflected light intensity, degree and angle of polarization for different microtextures and illumination conditions, we chose to plot the (normalized) intensity as a cartesian colourmap, with the horizontal (vertical) viewing direction φ(θ) as its *x*-axis (*y*-axis). The polarization state for each observer position is displayed by arrows, where the length of each arrow is proportional to the degree and angle of polarization can be extracted from the orientation of the arrow. For all observer positions (φ, θ), the orientation of the linearly polarized component of the incoming reflected light is displayed in the same way as a far away observer at (φ, θ) looking straight into the center of the illuminated microtexture would perceive it. In contrast to defining the propagation direction of the incoming light rays as the *z*-axis (optical axis) of the local coordinate system, as is common e.g. for linear polarizer filters, our methodology allows a direct comparison between the polarization orientations obtained by ray optical calculations and the experimentally determined polarization properties of light being reflected from microtextured surfaces. A graphical display of selected simulation results for the farfield intensity, degree and angle of polarization for all possible observer positions is given in S2 Fig As investigations into the properties of light being incoupled (instead of reflected) into such textures already demonstrated, the total power being reflected by these microtextures is being barely altered by both position and height disorder [1]. The general optical behaviour we experimentally obtained by imaging polarimetric analysis of PMMA rose petal replicas under direct sunlight (see Figs 4-6 and S1 Fig) is qualitatively reproduced by these purely ray optical numerical experiments. Direct quantitative comparisons between measured and calculated degree and angle of polarization yet have to be handled cautiously, since the simulations were restricted to illumination with parallel rays and therefore, the influence of the diffuse background illumination caused by scattering of sunlight in the earth’s atmosphere is not taken into account.

**Supporting reference**

[1] B. Fritz, R. Hünig, R. Schmager, M. Hetterich, U. Lemmer, G. Gomard, Assessing the influence of structural disorder on the plant epidermal cells’ optical properties: a numerical analysis, Bioinspiration & Biomimetics 12 (2017) 036011.
