# Supplementary material for: Bioreplicated coatings for photovoltaic solar panels nearly eliminate light pollution that harms polarotactic insects
Source: PLoS One. 2020 Dec 3;15(12):e0243296. doi: 10.1371/journal.pone.0243296 (PMC7714120; doi:10.1371/journal.pone.0243296)
Supplement: Attachment — Submitted filename: +RosePetalPol_PLoS-One_response.docx [file pone.0243296.s008.docx]

**Point-by-Point Response to the Comments of Referee 1**

We thank the constructive and valuable review of Referee 1. Our manuscript was revised on the basis of the reports of three Reviewers. All changes done with respect to the reviews of Referee 1, Referee 2 and Referee 3 are marked with green, orange and blue, respectively. Below is our detailed Point-by-Point Response to the comments of Referee 1.

**Referee 1 wrote**: *The English and overall writing of the manuscript is quite good, however, I had a very difficult time getting through the manuscript due to all of the abbreviations and jargon. For a manuscript in PLOS, the writing style should be accessible to the emerging scientist (i.e. undergraduate student) and non-scientist (preferably at a high school level) and unfortunately this paper does not meet that requirement. My partner, whom is a journalist, couldn't get through the first paragraph without looking stuff up or asking very specific questions. So, please rewrite the introduction - at least the first few paragraphs - to be more accessible to PLOS readers.*

*I think a more welcoming first couple of paragraphs in the introduction will drastically drive up the number of people who read the manuscript.*

**Answer**: We have rewritten and shortened the Introduction in order to be more accessible to the general reader. The criticized abbreviations were also omitted.

**Referee 1 wrote**: *Also, get rid of most of the abbreviations. I don't understand why you have an abbreviation for polarized light pollution or rose petal - just write it out. The abbreviation comes across as lazy and does not help with the manuscript. I would suggest getting rid of all of the abbreviations except for the chemical and UV, Vis abbreviations. I was frustrated by the abbreviations and how it was written for people only with a PhD in optics or visual sciences.*

**Answer**: In our revised manuscript the abbreviations AoI, AoP, AR, DoLP, MLA, NIR, PLP, PV, SEM, UV, Vis were replaced with their full meaning, and only the following abbreviations were kept in some cases:

CIGS: copper indium gallium diselenide, Cu(In,Ga)Se_2_

GRP: glass-covered rose petal

PMMA: polymethylmethacrylate

RP: rose petal

SBP: smooth black plastic

Brewster’s angle = arctan(*n*) from the normal vector of the reflecting surface, where *n* is the refractive index of the reflector’s material.

**Referee 1 wrote**: *I think you should use solar panel somewhere in your title or keywords so that it comes up if people don't search photovoltaic.*

**Answer**: (i) Our coatings are not only nanostructured, but also microstructured. (ii) ’bioreplicated’ is more correct than ’biomimetic’ in the present case. (iii) ’photovoltaic solar panels’ should be used to avoid possible confusion with heating of fluid by solar energy. Thus, we have modified our title as follows:

Bioreplicated coatings for photovoltaic solar panels nearly eliminate light pollution that harms polarotactic insects

The following was added to the keywords: solar panel

**Referee 1 wrote**: *The figures are well done but I am wondering if there is a way to add to figure 3 to show the amount of polarization? Could you add the mean polarization in figure 3B for each panel? It's difficult to compare between the 5 panels in each photo and to tell the actually difference in polarization.*

**Answer**: As required, the numerical values of the average degree of linear polarization were added to the revised Figure 2:

**Fig 2. Imaging polarimetry of the test surfaces.** Polarization patterns of 10 cm × 10 cm CIGS solar modules equipped with various cover layers under clear sky and for different observer viewing directions (columns): 1) Rose petal, 2) artificial microlens array, 3) planar PMMA, 4) no coating. For the cases of sun shining from in front of the observer, from behind the observer, from the left and from the right, three types of images are displayed: Colour photograph (top row), as well as degree of linear polarization *d* (middle row) and angle of polarization α (bottom row) at 450 nm (blue). In the middle row, the numerical values are the degrees of polarization averaged for the different test surfaces. The white bars in the bottom row show the local average direction of polarization. The tilt angle of the polarimeter’s optical axis was set to -35° from the horizontal.

**Referee 1 wrote**: *In the discussion it would be good to discuss a little more about the experimental implications of the mayflies and horseflies. I am interested in hearing the authors thoughts on whether these will greatly reduce attraction if the rose petaled panels are by themselves and the insects don't have surfaces that are more polarized? It makes sense that the insects will go to the more polarized surface, which could be the case if the only surface is the rose petal surface. Please discuss this more in each of the two insect sections in section 3.*

**Answer**: To the revised subsection *3.3. Attractiveness of Test Surfaces to Mayflies* we added the following:

In a pilot experiment we used only the rose-petal-replica matte black test surface, which however, did not attract any mayflies due to its low polarizing ability (see Fig 6). Therefore, we continued our field experiment with the use of two additional control surfaces that polarize the reflected light much stronger. From the pilot experiment we conclude that the rose petal replica reduces significantly the polarized light pollution for mayflies, even if it stands alone.

To the revised subsection *3.4. Attractiveness of Test Surfaces to Horseflies* we added the following:

Similarly to the experiment with mayflies, in the horsefly experiment we first presented only the rose-petal-replica matte black test surface, which however, did not attract any horsefly due to its low polarizing ability and because in sunshine it reflected not always horizontally polarized light (Figs 2, 4, S1 Fig). Thus, the alone-standing rose petal replica reduces significantly the polarized light pollution for horseflies, too. Therefore, we added two more polarizing control surfaces in the continuation of the horsefly experiment.

**Point-by-Point Response to the Comments of Referee 2**

We thank the constructive and valuable review of Referee 2. Our manuscript was revised on the basis of the reports of three Reviewers. All changes done with respect to the reviews of Referee 1, Referee 2 and Referee 3 are marked with green, orange and blue, respectively. Below is our detailed Point-by-Point Response to the comments of Referee 2.

**Referee 2 wrote**: *1. the title is very technical and does not advertise the biological aspect of the article. I suggest to modify e.g. to "Nanostructured coatings for solar panels nearly eliminate light pollution that harms polarotactic insects" or "Biomimetic nanostructured coatings..."*

**Answer**: (i) Our coatings are not only nanostructured, but also microstructured. (ii) ’bioreplicated’ is more correct than ’biomimetic’ in the present case. (iii) ’photovoltaic solar panels’ should be used to avoid possible confusion with heating of fluid by solar energy. Thus, we have modified our title as follows:

Bioreplicated coatings for photovoltaic solar panels nearly eliminate light pollution that harms polarotactic insects

**Referee 2 wrote**: *2. L426 and elsewhere - you talk about the DoP threshold of polarotactic aquatic insects, could you please specify some numbers or at least provide citations?*

**Answer**: These requested informations were presented in our original manuscript and highlighted with orange in our revised paper in the following ways:

1) We have written in the original Introduction the following:

If, for viewing directions near the Brewster’s angle the degree of polarization of reflected light is lower than the threshold of polarization sensitivity and/or if the direction of polarization of reflected light deviates from the horizontal by more than a given threshold value (both thresholds depend on the species considered), a polarotactic insect does not get attracted by the surface-reflected polarized light [29].

Reference [29] is an adequate citation:

[29] G. Kriska, B. Bernáth, R. Farkas, G. Horváth, Degrees of polarization of reflected light eliciting polarotaxis in dragonflies (odonata), mayflies (ephemeroptera) and tabanid flies (tabanidae), Journal of Insect Physiology 55 (2009) 1167-1173.

2) Reference [29] has been cited in the original subsection *3.2. Reflection-Polarization Characteristics* as follows:

In this case the angle of polarization is surely irrelevant, because the degree of polarization likely falls below the polarization sensitivity threshold of polarotactic insects ([29]), furthermore it may also fall within the uncertainty range of the sensor noise of our imaging polarimeter.

3) In the original subsection *3.5. Numerical Assessment of Reflection-Polarization Patterns of Microtextured Surfaces*, the DoP and AoP thresholds of polarotactic aquatic insects were specified and their citation was performed in the following way:

As reported in [29], surfaces reflecting light with a degree of polarization *d* > 15% and a polarization direction deviating by maximum ±10^o^ from the horizontal cause a maladaptive attractiveness to mayflies and horseflies because of misidentification as a water surface. These thresholds can be exploited to quantify the polarized light pollution (and its dependence on several structural parameters) that is caused by the reflection of direct sunlight from microtextured surfaces.

**Referee 2 wrote**: *3. The difference between DoP of surfaces, imaged during horsefly and mayfly experiments, is quite striking. I had to wait until the tables in the Supplement to get an explanation. Mayflies, who experienced DoP > 90%, were counted at the sunset, horseflies at around noon. At sunset the skylight is strongly polarized and it was the only illuminant of the panels. So DoP of reflections must be a consequence of complex interactions between the object properties and natural illuminant. If you agree, then please clarify this in the text.*

**Answer**: As suggested, to the revised subsection *3.2. Reflection-Polarization Characteristics* we added the following:

The differences between the degrees of polarization of test surfaces used in the experiments with horseflies (Fig 4) and mayflies (Fig 6) are quite striking, because mayflies were studied near sunset under twilight, while horseflies were investigated in full sunshine. At sunset the skylight and light reflected from the surrounding vegetation were the only light sources, while in sunshine the direct sunlight dominated. Thus, the reflection-polarization characteristics of the test surfaces are the consequence of complex interactions between the surface properties and the natural illumination conditions.

**Referee 2 wrote**: *4. The artificial surfaces are always compared to water surfaces. Although it may seem trivial, I regret that water was not imaged at the time of the experiments - due to the above mentioned complex interactions with the illuminant. Of course there are so many types of water, and these measurements should be repeated on the same place around the same time in the year and the day, but I really do not require further experiments. I am sure water has been measured and modeled many times, so please try to be quantitative and specify the values for DoP/AoP of water.*

**Answer**: As requested, in our revised manuscript we gave typical quantitative values of the degree and angle of polarization of water-reflected light and/or cited references containing such data as follows:

1. Introduction

From the Brewster’s angle, water surfaces reflect light with typical degrees of linear polarization 15 % ≤ *d* ≤ 90% and angles of polarization -10^o^ ≤ α ≤ +10^o^ relative to the horizontal direction, depending on their brightness/darkness, colour and surface roughness [20, 21, 22, 23, 29, 30, 31, 32, 33]. Water surfaces usually reflect nearly horizontally polarized light [33]. Aquatic insects in general (belonging to the following orders with aquatic or semiaquatic species: Coleoptera, Collembola, Diptera, Ephemeroptera, Hemiptera, Hymenoptera, Lepidoptera, Mecoptera, Megaloptera, Neuroptera, Odonata, Plecoptera, Trichoptera) have therefore evolved to identify water bodies by the horizontal polarization of water-reflected light [22, 23]. This strategy can result in a maladaptive attraction of polarotactic aquatic insects to smooth artificial surfaces like the glass/plactic covers of solar panels, because these surfaces can reflect a similar polarization pattern as water surfaces [23, 30, 31].

3.3. Attractiveness of Test Surfaces to Mayflies

The main reason for these findings can be explained by the facts that (i) *Ephemera danica* detects water with the horizontal polarization of water-reflected light [20, 32], (ii) the higher the degree of horizontal polarization of surface-reflected light, the more attractive the respective surface is to polarotactic mayflies [20, 29], and (iii) the degree of horizontal polarization of light reflected from our test surfaces decreased in the order: SBP > GRP > RP (see Fig 6).

3.4. Attractiveness of Test Surfaces to Horseflies

Since an approaching insect will always observe a targeted surface from multiple viewing angles and can track the change in polarization direction during its flight, a surface that only produces horizontal polarization for a narrow range of viewing directions is unlikely to be confused as a body of water [22, 23].

3.5. Numerical Assessment of Reflection-Polarization Patterns of Microtextured Surfaces

As reported in [29], surfaces reflecting light with a degree of polarization *d* > 15% and a polarization direction deviating by maximum ±10^o^ from the horizontal cause a maladaptive attractiveness to mayflies and horseflies because of misidentification as a water surface. These thresholds can be exploited to quantify the polarized light pollution (and its dependence on several structural parameters) that is caused by the reflection of direct sunlight from microtextured surfaces.

Furthermore, on the basis of numerous field experiments [e.g. 20, 21, 27, 28, 29, 31, 34], we assume that none of the possible observer viewing directions is favoured by the insects for spotting water surfaces.

**Point-by-Point Response to the Comments of Referee 3**

We thank the review of Referee 3. Our manuscript was revised on the basis of the reports of three Reviewers. All changes done with respect to the reviews of Referee 1, Referee 2 and Referee 3 are marked with green, orange and blue, respectively. Below is our detailed Point-by-Point Response to the comments of Referee 3.

**Referee 3 wrote**: *Lines 58/59: 'were almost unattractive to these species, and thus greatly reduced PLP'. The logic of this sentence seems wrong, or the statement exaggerated, in my opinion. It should be: 'were almost unattractive to these species, which is indicative of reduced PLP'.*

**Answer**: This sentence was revised as suggested:

By conducting field experiments with polarotactic horseflies (Diptera: Tabanidae) and a mayfly species (Ephemeroptera: *Ephemera danica*), we demonstrate that bioreplicated cover layers (here obtained by directly copying the surface microtexture of rose petals) were almost unattractive to these species, which is indicative of reduced polarized light pollution.

**Referee 3 wrote**: *Lines 223/224: Why was transmission not measured? Does the data exist?*

**Answer**: For polarotactic insects only the reflection(-polarization) characteristics of our test surfaces are important, rather than the transmission characteristics. Therefore, we measured only the former. In principle, the spectrum *T*(λ) of transmitted light could be calculated from the spectrum *I*(λ) and *R*(λ) of the incident and reflected light, respectively, assuming that *I*(λ) = *R*(λ) + *T*(λ), from which the transmission is: *T*(λ) = *I*(λ) - *R*(λ). However, this was irrelevant for our study.

**Referee 3 wrote**: *Lines 310-312: Polarimetric images of the asphalt would have helped making that claim.*

**Answer**: In Fig. 6 one can clearly see that the degree of polarization of asphalt-reflected light was much lower than that of test-surface-reflected light. In the revised subsection *2.5. Field Experiments with Mayflies* we wrote:

Since the degree of polarization of asphalt-reflected light was much lower than that of test-surface-reflected light (see Fig 6), furthermore the asphalt surface below and near the test surfaces was homogeneous, the asphalt did not affect the overall attractiveness to polarotactic mayflies.

**Referee 3 wrote**: *Lines 326 and 335/336: It's a bit weak that one has to trust an 'experienced observer', instead of having photographic/movie evidence. In the future, I recommend documenting these observations, in order to reduce personal bias.*

**Answer**: You are right. However, on the one hand, the continuous photographying of the test surfaces onto which tabanids landed would have disturbed their behaviour. On the other hand, the tabanid species *Tabanus tergestinus*, *T. bromius*, *T. bovinus*, *T. autumnalis*, *Atylotus fulvus*, *A. loewianus*, *A. rusticus* and *Haematopota italica* that occurred in the study site are well-known by the observer (G. H.) who has performed numerous field experiments with these species [21, 24, 25, 26, 27, 28, 29, 31, 34].

**Referee 3 wrote**: *Lines 364-366: Referring to unpublished and unprovided data is weak. One sentence explaining this claim would be helpful.*

**Answer**: In the revised subsection *2.7. Ray-Tracing Simulations of Reflection-Polarization Characteristics* we wrote:

Although sharply tipped cones do not fully follow the actual morphology of rose petal epidermal cells, our previous experimental and numerical analyses indicated that such idealized cones nevertheless allow to approximate the angle-dependent reflectance behaviour of petal texture replicas. Our previous simulation efforts on microtextures based on cones, ellipsoids and pyramids (not reported here) further showed that the general conclusions drawn from the simulations herein would not be affected by slight changes in the texture morphology, since all interactions are solely governed by ray-optical effects, which tend to be more robust against slight structural variations (in contrast to wave-optical effects like interference).
